# A Load-Balancing-Aware Learning Framework for Collaborative UAV-MEC Computation Offloading

**DOI:** 10.3390/s26061920

**Published:** 2026-03-18

**Authors:** Huafeng Li, Yuxuan Wang, Hengming Liu, Jiaxuan Li, Xu Wang, Qun Lei, Ke Xiao, Hongliang Zhu

**Affiliations:** 1 School of Electrical and Control Engineering, North China University of Technology, Beijing 100144, China; 2022413010117@mail.ncut.edu.cn; 2 School of Artificial Intelligence and Computer Science, North China University of Technology, Beijing 100144, China; wangyuxuan@mail.ncut.edu.cn (Y.W.);; 3 School of Cyberspace Security, Beijing University of Posts and Telecommunications, Beijing 100876, China

**Keywords:** UAV-MEC, computing task offloading, reinforcement learning, multi-objective optimization

## Abstract

Unmanned Aerial Vehicle (UAV) computing clusters face severe operational constraints due to limited computing capabilities and battery capacities, which complicate the simultaneous optimization of low offloading latency, long task endurance, and high cluster efficiency. To address these challenges, this paper proposes a Multi-Objective Reinforcement Learning framework based on Latency and Power Balance (MORL-LAPB). Instead of broad situational awareness descriptions, our framework directly combines a reward-shaping reinforcement learning algorithm with an evolutionary mechanism to construct a closed-loop optimization paradigm. Crucially, in this context, ’balancing’ extends beyond traditional computational workload distribution; it represents a joint optimization that balances task allocation to ensure short service delays while simultaneously equating the energy depletion rates across UAV nodes to maximize overall cluster efficiency and operational duration. By efficiently identifying Pareto optimal trade-offs, MORL-LAPB dynamically regulates UAV energy allocation and computational resource scheduling. Experimental results demonstrate that, compared to RSO, NSO, and DRLSO baselines, the proposed MORL-LAPB significantly reduces offloading latency, extends effective task execution duration, and improves cluster energy efficiency. The framework offers flexible adaptability and long-term sustainability for diverse operational scenarios under strict multi-objective constraints.

## 1. Introduction

**Figure 1:**
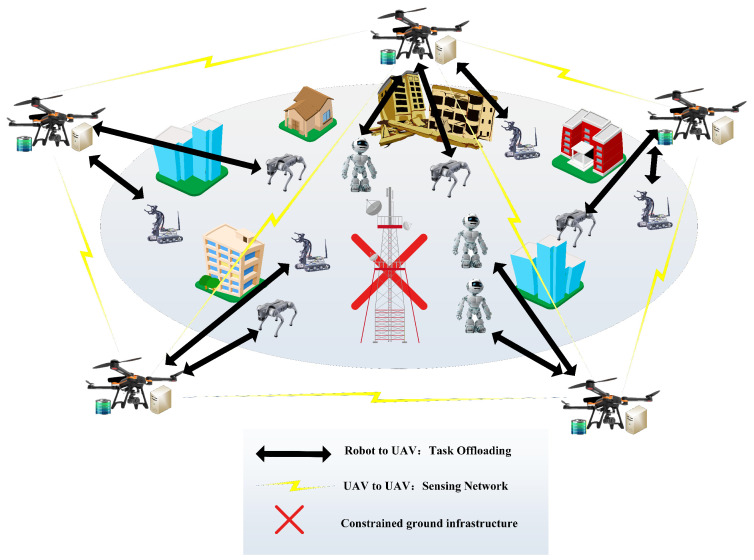
Application of UAV-MEC in infrastructure-weak areas.

Unmanned Aerial Vehicle-assisted Mobile Edge Computing (UAV-MEC) clusters serve as critical intelligent resources in regions with underdeveloped infrastructure, where traditional fixed ground edge servers often fail to provide effective computing services [1,2,3,4]. As illustrated in Figure 1, multiple UAV-MECs form a collaborative Computing-aware Network (CAN) to assist ground intelligent agents in executing computation-intensive missions (e.g., semantic understanding and object recognition) [5,6]. Upon receiving tasks, each UAV-MEC node makes intelligent decisions to execute a portion of the workload locally while offloading the remainder to other UAV-MECs via wireless links for parallel processing [7,8].

Despite their high mobility and flexible deployment capabilities [9,10], the sustained operation of UAV-MEC clusters is heavily restricted by the inherent bottlenecks of onboard battery capacities and computing capabilities. This creates a severe multi-objective optimization challenge involving competing demands: guaranteeing low task offloading latency for real-time responsiveness, maximizing the operational endurance of the cluster, and improving overall energy efficiency.

To address these conflicting objectives simultaneously, effective load-balancing is paramount. In our proposed framework, load-balancing extends beyond the conventional even distribution of computational workloads. Rather, it serves as the core mechanism that tightly couples energy consumption and task delays. By dynamically balancing the task load among UAVs, the system prevents individual nodes from becoming computational bottlenecks—thereby minimizing queuing delays (reducing latency). Simultaneously, load-balancing equalizes the energy depletion rates across the cluster, preventing premature node failure and maximizing both the effective operational duration and cluster energy efficiency. Therefore, achieving a latency and power-aware load balance is the fundamental key to overcoming the operational constraints of UAV-MEC systems.

Although traditional heuristic algorithms (e.g., genetic algorithms, particle swarm optimization) and conventional non-learning scheduling baselines perform well in static or small-scale problems, they exhibit significant limitations in the highly dynamic and high-dimensional scheduling scenarios inherent to UAV-MEC networks. First, they demonstrate insufficient adaptability. Traditional heuristics rely on fixed rules or offline manual feature designs. When environmental parameters fluctuate, they lack online sensing capabilities and cannot leverage historical experience to accelerate decision-making. Second, conventional heuristics and simple machine learning methods are inherently short-sighted and reactive. They make greedy decisions based solely on the immediate state. However, in UAV-MEC computation offloading, current decisions dictate future system blocking rates and throughput. By ignoring the correlation between immediate actions and long-term cumulative future rewards, these traditional methods frequently fall into local optima.

To overcome these limitations, we propose a novel Multi-Objective Reinforcement Learning framework based on Latency and Power Balance (MORL-LAPB). The motivation behind this design is to transition from reactive heuristics to a forward-looking, self-learning system. Through continuous environmental interaction, MORL-LAPB automatically extracts hidden patterns without relying on rigid heuristic rules. It resolves objective conflicts by seamlessly co-optimizing latency, operational duration, and cluster efficiency within a single model. Crucially, by leveraging a reinforcement learning reward mechanism, MORL-LAPB grounds its sequential decisions in long-term cumulative rewards, achieving true global optimality rather than settling for short-sighted, localized gains.

The remainder of this paper is organized as follows. Section 2 reviews related works. Section 3 presents the system model and formulates the multi-objective problem. Section 4 details the proposed MORL-LAPB framework. Section 5 evaluates the performance through simulation studies, followed by a comprehensive discussion in Section 6. Finally, Section 7 concludes the paper.

## 2. Related Work

In this section, we review the literature on UAV-MEC computation offloading. Early research addressed complex UAV network challenges—such as trajectory optimization and multi-hop multicasting—primarily using traditional mathematical methods like convex optimization and game theory. While offering rigorous theoretical bounds, these non-ML approaches rely heavily on static models and face prohibitive computational complexity in highly dynamic, multi-objective environments. To overcome these limitations and achieve real-time adaptability, recent studies have increasingly transitioned to Reinforcement Learning (RL). Accordingly, we categorize the existing literature based on their primary optimization objectives: latency, energy efficiency, and joint optimization.

### 2.1. Latency Optimization for Computation Offloading

To meet the low-latency requirements of computation-intensive tasks, researchers have focused on optimizing offloading and scheduling strategies. Li et al. [11] proposed an online task partitioning and cooperative offloading method for dual edge servers (ESs), which dynamically distributes workloads between two ESs for parallel processing, effectively reducing processing latency. Similarly, Xu et al. [12] further explored the potential of parallel processing in heterogeneous MEC environments. Their proposed SMCoEdge method dynamically selects multiple ESs and allocates workloads to fully utilize idle computing resources, thereby accelerating task completion.

In more dynamic UAV scenarios, latency optimization faces greater challenges. Tang et al. [13], considering UAV mobility and the dynamic variation of network traffic, designed an improved double Q-learning algorithm that enables UAVs to make decisions based on local and neighboring historical information, significantly reducing transmission latency and packet loss. Liu et al. [14] focused on aquatic environments and constructed a two-tier communication network consisting of centralized upper-layer UAVs and distributed lower-layer UAVs. They formulated the joint minimization of communication and computation delay as a Markov Decision Process (MDP) and employed deep reinforcement learning algorithms such as DQN and DDPG to optimize the trajectories of upper-layer UAVs, achieving global latency optimization from a network architecture perspective.

These studies primarily address latency challenges through multi-ES collaboration, parallel processing, and intelligent trajectory planning. However, many works focus on optimizing a single performance metric, without fully accounting for the associated energy consumption overhead.

### 2.2. Energy-Efficient Optimization for Computation Offloading

Due to the limited onboard energy of UAVs, energy consumption has become a key optimization objective. Most studies in this area aim to achieve energy efficiency by jointly optimizing UAV trajectories along with communication and computation resources. In a single-UAV multi-user scenario, Wang et al. [15] minimized UAV energy consumption by decomposing the problem and jointly optimizing task offloading and flight trajectory. Building upon this, Liu et al. [16] introduced user cooperation and wireless power transfer (WPT) technology to jointly optimize computation frequency, offloaded data volume, and flight trajectory, further expanding the potential for energy savings. To address the high complexity of centralized decision-making, Pervez et al. [17] modeled multi-user offloading decisions in an air–ground collaborative environment as a potential game, deriving the optimal distributed strategy by finding the Nash equilibrium, which effectively minimized the system’s weighted total cost.

In recent years, reinforcement learning (RL) has demonstrated strong potential to tackle such complex optimization problems due to its ability to handle high-dimensional continuous state spaces. Wang et al. [18] proposed an online trajectory optimization method based on an actor–critic (AC) framework to adapt to dynamic environments where UAVs take off from different locations. Chen et al. [19] further incorporated the Age of Information (AoI) metric and applied a double delayed deep Q-network (D3QN) model to minimize device energy consumption while maintaining data freshness.

Energy-efficient optimization research has evolved from traditional convex optimization approaches to more advanced methods combining game theory and reinforcement learning to address complex decision-making in dynamic environments. However, an excessive focus on minimizing energy consumption may come at the expense of task processing speed.

### 2.3. Joint Optimization of Latency and Energy Consumption

In practical applications, latency and energy consumption are often conflicting performance indicators. Therefore, how to jointly optimize both to maximize overall system efficiency has become a key research focus in recent years.

Gao et al. [20] employed game theory to model the interactions between multiple users and UAVs, adopting the Multi-Agent Deep Deterministic Policy Gradient (MADDPG) method to optimize UAV trajectories. Their approach ensured obstacle avoidance while balancing user-side latency and energy efficiency. Wu et al. [21] treated UAVs as aerial base stations serving ground vehicles and proposed an RL-based deployment strategy to predict traffic conditions and determine optimal UAV hovering positions, aiming to reduce flight and turning energy consumption while maintaining service continuity. Sun et al. [22] presented a more comprehensive framework—the Joint Task Offloading and UAV Trajectory Control (JTOUTC) algorithm—which integrates block coordinate descent and successive convex approximation techniques to simultaneously minimize task completion latency and UAV energy consumption, while maximizing the volume of offloaded tasks.

These methods have achieved promising results by incorporating trajectory planning, transmission power allocation, and CPU frequency control. However, most existing studies focus on minimizing the energy consumption of individual UAVs, lacking a holistic consideration of computation offloading and overall energy efficiency at the UAV-MEC cluster level.

### 2.4. Research Gaps and Proposed Positioning

Recently, Multi-Objective Reinforcement Learning (MORL) has gained widespread attention for balancing conflicting metrics, such as latency and energy consumption, in MEC environments. However, most existing MORL approaches rely on static linear scalarization to combine these objectives, which struggles to adaptively address the dynamic energy variance—or energy balancing—across distributed UAV nodes. Energy balancing is particularly critical in distributed MEC to prevent the “wooden barrel effect,” where the premature battery depletion of a single heavily loaded node paralyzes the entire cooperative cluster. While traditional methods attempt to mitigate this through heuristic load distribution, they lack the adaptive and far-sighted capabilities inherent to RL.

Compared to existing research, this work distinguishes itself through three core innovations:

1. **Optimization Paradigm Shift:** While existing methods predominantly aim to “minimize total cluster energy consumption” or “individual node energy consumption,” we expand the optimization objective to “minimize the residual energy variance of the cluster.” This fundamental shift directly addresses the critical issue of cluster energy balancing that traditional methods overlook.
2. **Algorithmic Architecture Breakthrough:** Breaking through the limitations of static linear scalarization in traditional MORL, we propose a hybrid Evolutionary-MORL framework. The outer evolutionary algorithm dynamically searches for Pareto-optimal weights, while the inner DRL agent trains policies accordingly, achieving an adaptive optimal trade-off between latency and energy consumption within a non-convex solution space.
3. **Physics-Informed Integration Mechanism:** We design a physics-informed reward shaping mechanism that embeds the energy balance deviation ($\partial_{t}$) as a regularization term into the reward function. This mechanism strictly guides the RL agent to autonomously learn load-balancing strategies during the dynamic decision-making process.

### 2.5. Comparative Analysis

As synthesized in Table 1, the existing literature has generally evolved from traditional mathematical models [15,16,17] to learning-based dynamic decision frameworks [13,14,18,19,20,22]. While the former guarantees optimality in static settings and the latter enables real-time adaptability, a critical gap remains: current multi-objective approaches predominantly focus on minimizing overall or individual energy costs, systematically ignoring the equitable balance of energy distribution across the cluster.

To bridge this specific gap, our proposed MORL-LAPB introduces a hybrid Evolutionary-MORL framework. By explicitly formulating energy balancing as a core objective alongside latency reduction, and automating hyperparameter tuning via an evolutionary algorithm, the framework conducts an adaptive Pareto-optimal search. This fundamentally prevents the premature depletion of individual UAVs (the “wooden barrel effect”), ensuring robust and sustainable cluster coordination.

## 3. System Model and Problem Formulation

This section first introduces the proposed system model and then formulates the corresponding optimization problem.

### 3.1. System Model

To fully exploit the overall performance of the UAV-MEC system and validate the effectiveness of the proposed method, we consider a multi-UAV collaborative edge computing cluster consisting of *B* UAV-MECs, denoted as B={1,…,B}, where *b* represents the *b*-th UAV-MEC in the cluster and satisfies b∈B.

Each UAV is equipped with a mobile edge computing (MEC) server powered by an onboard battery and collaborates with other UAVs via a wireless communication network. Different MEC servers possess heterogeneous computing capacities *f* and battery energy states $C_{t}$. The computing capacity *f* depends on the type of UAV-MEC processor and its operating frequency, while the battery energy state $C_{t}$ is jointly determined by the battery capacity $C_{f}$, the dynamic power consumption $Q_{d}$ of the computing unit, and the static power consumption $Q_{s}$ of the computing unit. For ease of reference, the key symbols used throughout this paper are summarized in Table 2.

User terminals act as computation consumers and continuously generate data and issue offloading requests N. For analytical convenience, continuous time is discretized into time slots of unit length. The total scheduling duration *T* is represented as a set of discrete time slots, denoted by T={1,…,T}, where t∈T denotes the *t*-th time slot. Within the same time slot, multiple offloading tasks from different user terminal groups are aggregated and assigned to various UAV-MECs.

Taking the time slot *t* at which a UAV-MEC receives tasks as the reference, we define $N_{b,t}$ as the set of offloading tasks received by UAV-MEC *b* during time slot *t*, i.e., $N_{b,t}=\left\{1,2,\ldots ,N_{b,t}\right\}$, where *n* denotes the index of an offloading task in this set.

Each computation offloading task is characterized by a triplet $\left(d_{n},\rho_{n},\tau_{n}\right)$, where $d_{n}$ (in bits), $\rho_{n}$ (in CPU cycles per bit), and $\tau_{n}$ (in seconds) represent the task data size, computational intensity, and deadline, respectively.

Each UAV-MEC is equipped with a scheduler and an executor. The scheduler is responsible for task reception, parsing, decomposition, and subtask allocation. Specifically, when a UAV-MEC receives a computation task from a user terminal, the scheduler first identifies the task type and requirements, then parses and decomposes the task to determine the corresponding subtask proportions. Finally, the scheduler makes subtask offloading decisions by comprehensively considering the requirements of all computation tasks and the current state information of UAV-MECs within the cluster.

Similarly to existing studies, we define the decision variable $x_{b,n,t,b^{\prime }}\in \left\{0,1\right\}$ to indicate whether task *n*, received by UAV-MEC *b* in time slot *t*, is assigned to UAV-MEC $b^{\prime }\in B$ for processing. Accordingly, the constraint ensuring that the sum of allocated decisions equals one is formulated in Equation (1) as follows:

(1) $$\sum_{b^{\prime }\in B}x_{b,n,t,b^{\prime }}=1,\;\forall b\in B,\;n\in N_{b,t},\;t\in T.$$

The executor is responsible for the management and execution of subtasks. Upon receiving the subtasks decomposed by the scheduler, it buffers them in a task queue based on their arrival order and processes them in a first-in-first-out (FIFO) manner. Let $T_{n,t,b^{\prime }}^{o}$ denote the processing latency of task *n* offloaded to UAV-MEC $b^{\prime }$ during time slot *t*, and let $T_{n,t}^{s}$ represent the total completion time (make-span) of that task. If the task cannot be completed within its deadline $\tau_{n}$, it is deemed an offloading failure, and the system immediately discards the task.

**Figure 2:**
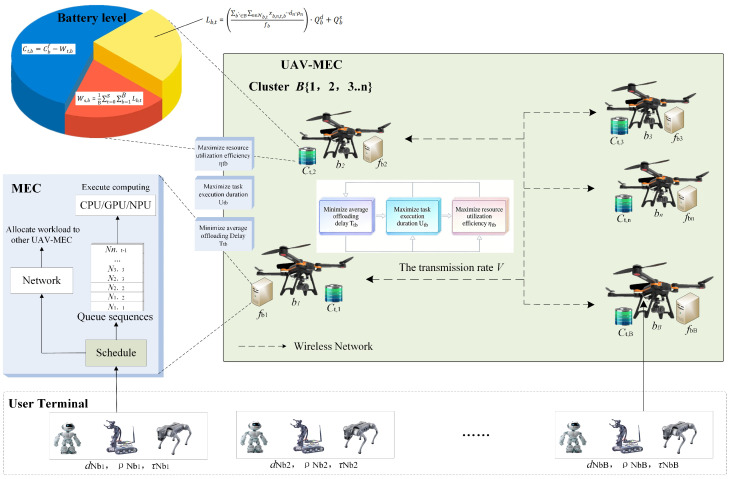
System model of a cooperative UAV computing cluster.

The task execution process incurs energy consumption $L_{b,t,b^{\prime }}$. When the battery energy state $C_{t}$ of any UAV-MEC in the cluster drops to zero, the total accumulated working time $T_{d}$ at that moment is defined as the operational duration of the UAV-MEC cluster. To clearly illustrate the architecture and operational interactions within the proposed cooperative UAV computing cluster, the system model is depicted in Figure 2.

#### 3.1.1. Offloading Delay Model

Let $T_{b,n,t,b^{\prime }}^{o}$ denote the processing delay of task *n* that arrives at UAV-MEC *b* at time slot *t* and is offloaded to UAV-MEC $b^{\prime }\in B$. The total processing delay consists of three components: the transmission delay from UAV-MEC *b* to UAV-MEC $b^{\prime }$, the computation delay incurred at UAV-MEC $b^{\prime }$, and the queuing delay in the task buffer of UAV-MEC $b^{\prime }$. Consequently, the overall processing delay can be expressed as

(2) $$T_{b,n,t,b^{\prime }}^{o}=x_{b,n,t,b^{\prime }}\left(\frac{d_{n}}{v_{b,b^{\prime },t}}+\frac{d_{n}\rho_{n}}{f_{b^{\prime }}}+T_{b,n,t,b^{\prime }}^{w}\right)$$

where $v_{b,b^{\prime },t}$ denotes the transmission rate from UAV-MEC *b* to UAV-MEC $b^{\prime }$, $f_{b^{\prime }}$ represents the computing capability of UAV-MEC $b^{\prime }$, and $T_{b,n,t,b^{\prime }}^{w}$ denotes the queuing time of task *n* at UAV-MEC $b^{\prime }$ during time slot *t*, which is obtained through observing the system. In addition, let $T_{b,n,t}^{s}$ denote the completion time of task *n* that arrives at UAV-MEC *b* at time *t*. Then, $T_{b,n,t}^{s}$ is equal to the maximum processing delay among all workloads assigned to UAV-MEC *b*, or the deadline when the task is dropped, i.e., $T_{b,n,t}^{s}=\max_{b^{\prime }\in B}\left\{T_{b,n,t,b^{\prime }}^{o}\right\}$.

#### 3.1.2. Cluster Job Duration Model

In this system model, the energy consumption is closely related to computation task execution. Let $L_{b,t}$ denote the total energy consumption of UAV-MEC b∈B during time slot *t* for processing all received computation tasks. The cumulative energy consumption of UAV-MEC *b* from the start of task execution up to time slot *t* is denoted by $W_{t,b}$, and the residual battery energy state of UAV-MEC *b* at time slot *t* is denoted by $C_{t,b}$.

(3) $$L_{b,t}=\sum_{b^{\prime }\in B}\sum_{n\in N_{b,t}}x_{b,n,t,b^{\prime }}\cdot \frac{d_{n}\rho_{n}}{f_{b^{\prime }}}\cdot Q_{b}^{d}+Q_{b}^{s}$$

(4) $$W_{t,b}=\sum_{s=0}^{t}L_{b,s},\;t\in T$$

(5) $$C_{t,b}=C_{b}^{f}-W_{t,b}$$

where $Q_{b}^{d}$ denotes the dynamic energy consumption coefficient of the computing unit of UAV-MEC *b* during a time slot of length Δ, and $Q_{b}^{s}$ represents the static energy consumption of the computing unit within the same period.

If the residual battery energy of UAV-MEC *b* drops to zero at time slot $t_{b}$, i.e., $C_{t_{b},b}=0$, then the operational duration of the UAV-MEC cluster is defined as $t_{b}=\left\{\;t|C_{t,b}=0\;\right\}$.

#### 3.1.3. Cluster Energy Efficiency Model

When the cluster operation time satisfies $T_{d}=t_{b}$, indicating that all computation tasks within the UAV-MEC cluster have been completed, the remaining battery capacities of the UAV-MEC nodes can be represented as the set $C_{t_{b}}=\left\{C_{t_{b},1},C_{t_{b},2},\ldots ,C_{t_{b},B}\right\}.$ Accordingly, the cluster energy efficiency is defined as

(6) $$\eta =1-\frac{\sum_{b=1}^{B}C_{t_{b},b}}{\sum_{b=1}^{B}C_{b}^{f}}.$$

If the UAV-MEC cluster achieves maximum operational efficiency, the battery energy of all UAV-MEC nodes should ideally decrease to zero simultaneously upon task completion, i.e., $C_{t_{b},1}=C_{t_{b},2}=\ldots =C_{t_{b},B}=0.$ Similarly, at any given time slot *t*, the overall energy state of the cluster can be represented as a vector $C_{t}=\left\{C_{t,1},C_{t,2},\ldots ,C_{t,B}\right\}.$ We define the power balance deviation at time slot *t*, denoted by $\partial_{t}$, to quantify the degree of energy imbalance among UAV-MEC nodes within the cluster, which is expressed as $\partial_{t}=\frac{1}{C_{f}}\left(\max_{b\in B}C_{t,b}-\min_{b\in B}C_{t,b}\right)$.

### 3.2. Problem Formulation

The objective of this paper is to fully utilize the computing resources of the UAV cluster under certain cluster energy constraints, leveraging the advantages of the collaborative UAV cluster to provide low-latency services $T^{S}$, maximize the task execution duration of the UAV collaborative cluster $T_{d}$, and maximize the cluster energy efficiency $\eta_{t}$. To comprehensively evaluate these three performance dimensions, we define a joint global utility function J.

In summary, to maximize this global utility J, the UAV collaborative cluster task offloading problem can be formulated as the following online optimization problem:

(7a) $$\begin{array}{cc} \max_{x}\; & J\left(x\right)=\left(\eta_{t},T_{d},-T^{S}\right) \end{array}$$

(7b) $$\begin{array}{cc} s.t.\;\; & \sum_{b^{\prime }\in B}x_{b,n,t,b^{\prime }}=1,\;\forall b\in B,n\in N_{b,t},t\in T \end{array}$$

(7c) $$\begin{array}{cc} \; & x_{b,n,t,b^{\prime }}\in \left\{0,1\right\},\;\forall b\in B,n\in N_{b,t},t\in T \end{array}$$

(7d) $$\begin{array}{cc} \; & \eta_{t}\leq 1-\frac{\sum_{b=1}^{B}\left(C_{b}^{f}-\sum_{t=1}^{T_{d}}L_{b,t}\right)}{\sum_{b=1}^{B}C_{b}^{f}} \end{array}$$

(7e) $$\begin{array}{cc} \; & 0<T_{d}<\frac{C_{b}^{f}}{Q_{b}^{s}} \end{array}$$

(7f) $$\begin{array}{cc} \; & T^{S}\geq \frac{1}{\sum_{t=1}^{T_{d}}\left|N_{t}\right|}\sum_{t=1}^{T_{d}}\sum_{b=1}^{B}\sum_{n\in N_{b,t}}T_{b,n,t^{\prime }}^{S} \end{array}$$

where Equation (7a) represents the joint multi-objective function aimed at maximizing cluster energy efficiency $\eta_{t}$, operational duration $T_{d}$, and minimizing service delay (represented as maximizing −$T^{S}$). Constraints (7b) and (7c) define the valid domain for the binary task offloading decisions. Constraint (7d) defines the upper bound for the cluster energy efficiency $\eta_{t}$, derived from the residual energy at the termination moment. Constraint (7e) establishes the strict theoretical bounds for the operational duration $T_{d}$. Constraint (7f) enforces that the service delay indicator $T^{S}$ is bounded below by the average processing make-span of all admitted tasks.

The optimization problem presented in Equation (7a) is formulated over a non-convex feasible set, involving binary decision variables as defined in the constraints. This property classifies the optimization problem as a mixed-integer nonlinear programming (MINLP) problem, which is known to be NP-hard.

Similarly, the problem defined by Equation (7a) can be analogous to a multi-knapsack problem: each UAV-MEC is treated as a knapsack, with its capacity determined by the maximum load $f_{b}$. The goal is to assign as many tasks as possible—favoring those with shorter completion times and longer operational durations—into the UAV-MEC set *B* without exceeding the individual UAV weight capacities $f_{b}$. Since the knapsack problem with complex non-linear constraints is a well-known NP-hard problem, it follows that the constructed problem in Equation (7a) is also NP-hard. Moreover, in the UAV-MEC cooperative cluster scenario considered in this work, many decisions depend on the instantaneous states of various system components, which further complicates solving the problem using traditional optimization methods.

## 4. Solution Approach

In this section, we provide a detailed description of our proposed method and algorithm. In Section 4.1, we introduce the overall framework of our approach and elaborate on the key processes, including environment and state observation, reward mechanism design, offloading action decision-making, and parameter training and updating. Subsequently, in Section 4.2, we present the pseudocode and optimization design of the MORL-LAPB algorithm.

### 4.1. Method Framework

While existing works [13,14] utilize DRL for sensing-based offloading, they typically treat sensing information merely as state input to minimize cumulative system costs (e.g., total energy consumption). In contrast, our approach advances the algorithmic design from both objective and optimization perspectives. Table 1 summarizes the differences between our proposed method and several representative approaches in collaborative MEC.First, at the objective level, most existing studies primarily aim to minimize offloading latency while jointly reducing the overall energy consumption of UAV-MEC systems. Our method instead formulates a joint optimization objective that simultaneously minimizes offloading latency and the cluster power deviation, thereby explicitly promoting long-term energy balance among UAVs rather than merely reducing aggregate energy usage. This distinction shifts the optimization focus from system-level energy efficiency to sustainable cluster operation. Second, at the methodological level, prior works largely emphasize system-level energy optimization through trajectory planning, user scheduling, transmission power allocation, and CPU frequency control, forming a comprehensive yet predominantly energy-centric framework. In contrast, our method concentrates on computational task offloading objective allocation and introduces two key algorithmic innovations. (i) Rather than relying on passive sensing, we design a feedback-driven reward shaping mechanism in which the sensed energy variance $\left(\partial_{t}\right)$ directly modulates the gradient descent direction of the RL agent, explicitly steering policy updates toward energy-balanced states. (ii) We further embed the RL agent within an evolutionary optimization framework to automate hyperparameter tuning, replacing the static reward structures commonly adopted in the related literature with a dynamic and adaptive weighting mechanism. More fundamentally, our framework establishes a multi-perspective paradigm for intelligent computation offloading in UAV-MEC environments, integrating latency-aware scheduling, energy deviation regulation, and adaptive learning dynamics into a unified optimization architecture.

The optimization problem formulated in Section 3 is a mixed-integer nonlinear programming (MINLP) problem with hard constraints, which is generally intractable for real-time decision-making under uncertainty. To make the problem solvable via RL, we relax the hard constraints and incorporate them into the reward function as penalty terms. This transforms the original constrained optimization into an unconstrained goal of maximizing a scalar reward that heavily penalizes infeasibility. Although this relaxation does not offer a strict theoretical guarantee, the penalty coefficients are tuned such that the learned policy violates the original constraints (e.g., the QoS deadline constraint in Equation (7e)) with a negligible probability.

To tackle the aforementioned NP-hard problem and achieve efficient computation task offloading while maximizing both the operational duration and energy efficiency of the UAV cooperative cluster, this work employs a multi-objective reinforcement learning algorithm based on queueing-sequence task delay and energy balancing, termed MORL-LAPB, to optimize offloading decisions.

**Figure 3:**
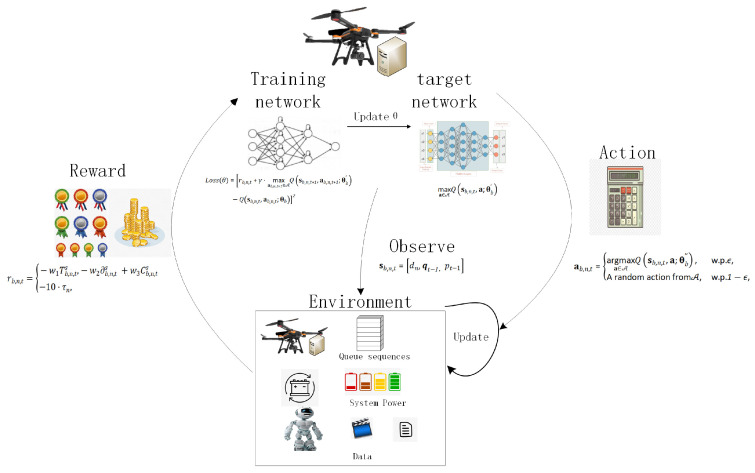
Energy-balanced UAV-MEC computation task offloading framework based on reinforcement learning.

MORL-LAPB is built on a deep reinforcement learning (DRL) architecture. To ensure cooperative behavior, the system adopts a Centralized Training with Decentralized Execution (CTDE) paradigm. During centralized training, the network aggregates global observations of all UAV task queues and energy states to learn cooperative policies. During decentralized execution, each UAV-MEC operates as an independent agent, deploying identical target networks in a distributed manner. Specifically, the target network observes the environment and cluster state to compute the corresponding offloading actions. Once an action is executed, the environment and cluster state are updated in real time, and the UAV-MEC receives the corresponding reward signal. Meanwhile, the training network collects historical offloading data for model training and periodically copies its parameters to update the target network. The overall framework is illustrated in Figure 3.

#### 4.1.1. Environment and State Observation

Each UAV-MEC receives offloading task requests N from user terminals, obtaining, for each task *n*, the data size $d_{n}$, computational intensity $\rho_{n}$, and deadline $\tau_{n}$. At each time step t∈T, every MEC observes the system’s state information, which primarily includes the task queue load of each UAV-MEC and the cluster’s energy status. Let $s_{b,n,t}$ denote the state information of UAV-MEC *b* while processing task *n* at time *t*, which can be represented as

(8) $$s_{b,n,t}=\left\{d_{n},q_{t-1},P_{t-1}\right\}$$

where $q_{t-1}=\left\{q_{t-1,1},\ldots ,q_{t-1,b^{\prime }},\ldots ,q_{t-1,B}\right\}$ denotes the load level of the UAV-MEC cluster at the end of time t−1. $q_{t,b}$ (in CPU cycles) represents the workload of the task queue for UAV-MEC *b* at time *t*, which is updated as

(9) $$q_{t,b}=\max\left\{\sum_{b\in B}\sum_{n\in N_{b,t}}x_{b,n,t,b}\cdot d_{n}\cdot \rho_{n}+q_{t-1,b}-f_{b}\Delta \right\},$$

where $P_{t-1}=\left\{P_{t-1,1},\ldots ,P_{t-1,b^{\prime }},\ldots ,P_{t-1,B}\right\}$ denotes the cumulative energy consumption of the UAV-MEC cluster at the end of time t−1. $P_{t,b}$ (in energy units) represents the energy consumed by UAV-MEC *b* at time *t*, and it is updated according to the following formula: $P_{t,b}=P_{t-1,b}+L_{b,t}$.

**Action Space:** For each time slot *t*, the offloading decision for each arriving task *n* involves selecting a target server from the available UAV-MEC cluster. Thus, the individual action for a single task can be defined as $a_{b,n,t}\in \left\{1,\ldots ,B\right\}$. Consequently, for a UAV-MEC node handling *N* tasks simultaneously within a time slot, the size of the joint action space is $O(B^{N})$. As the action space grows exponentially with the number of tasks *N*, finding an exact optimal solution using deterministic algorithms (such as dynamic programming) in polynomial time is strictly limited to extremely small-scale scenarios. This severe exponential complexity inherently dictates and justifies our adoption of a Deep Reinforcement Learning (DRL) approach, which aims to efficiently discover high-quality approximate solutions with an acceptable computational overhead.

#### 4.1.2. Reward Mechanism Design

The reward is central to the interaction between UAV-MEC and the environment, and designing a reasonable reward mechanism is crucial for the success of the entire learning process. After making offloading decisions in each episode, a UAV-MEC receives an offloading reward, with the goal of maximizing the cumulative task reward over the entire task period. Essentially, the reward reflects a predefined preference for certain actions and states. In the context of this method, it is designed to satisfy the expressions and constraints in Equation (7a), targeting low offloading delay, extended cluster operational time, and high utilization efficiency of cluster resources.

For the offloading delay requirement, as defined in the previous section, $T_{b,n,t}^{s}$ equals the longest processing delay among the workloads assigned to the UAV-MEC cluster. The cluster operational time cannot be determined directly, since the energy consumption for time steps t+1 and beyond is unknown at time *t*. Based on physical principles, assuming that higher remaining energy corresponds to longer UAV-MEC operation time, a larger $C_{b,n,t}^{s}$ implies a longer cluster operational time $T_{d}$, where b∈B can be any UAV-MEC in the cluster.

For the cluster efficiency requirement, an energy balancing strategy is adopted, i.e., $\lim_{t\to t_{b}}\partial_{b,n,t}=0$, which satisfies the constraint $C_{t_{b},1}=C_{t_{b},2}=C_{t_{b},3}=\ldots =C_{t_{b},B-2}=C_{t_{b},B-1}=C_{t_{b},B}=0$.

In summary, we select $T_{b,n,t}^{s}$, $C_{b,n,t}^{s}$, $\partial_{b,n,t}$ as the reward components corresponding to offloading delay, operational time, and cluster efficiency, respectively. A reinforcement learning reward mechanism is established based on offloading delay, energy balance, and residual energy to simultaneously achieve minimum offloading delay, maximum cluster operational time, and maximum cluster efficiency. This transforms the multi-objective computation task offloading problem into a single-objective offloading reward problem.

Let $r_{b,n,t}$ denote the reward of task *n* offloaded to UAV-MEC *b* at time *t*. Then, $r_{b,n,t}$ can be expressed as

(10) $$r_{b,n,t}=\left\{\begin{array}{cc} -w_{1}T_{b,n,t}^{s}-w_{2}\partial_{b,n,t}^{s}+w_{3}C_{b,n,t}^{s}, & \mathrm{if}\;\mathrm{delay}\;\mathrm{constraint}\;\mathrm{is}\;\mathrm{satisfied}\\ -w_{4}\cdot \tau_{n}, & \mathrm{if}\;\mathrm{delay}\;\mathrm{constraint}\;\mathrm{is}\;\mathrm{not}\;\mathrm{satisfied} \end{array}\right.$$

In this framework, the reward value is designed as a weighted combination of offloading latency, energy balance deviation, and residual energy. Specifically, $w_{1}$, $w_{2}$, and $w_{3}$ represent the weight parameters for these respective factors, which are dynamically adjusted based on the system’s requirements for offloading delay, cluster operational duration, and operational efficiency. The offloading latency and energy balance deviation serve as penalty terms, exhibiting a negative correlation with $r_{b,n,t}$; thus, larger values result in lower rewards. Conversely, the residual energy acts as a positive incentive, where higher values yield larger rewards. Furthermore, if an offloading task fails to meet the service delay constraint, it is deemed a failure and incurs a severe penalty, quantified by the penalty coefficient $w_{4}$. Unlike the dynamically tuned multi-objective weights ($w_{1},w_{2},w_{3}$), $w_{4}$ is set to a fixed parameter (e.g., $w_{4}=10$) based on our prior empirical knowledge and preliminary experiments. While a dynamic penalty mechanism presents an interesting avenue for optimization, the fixed-weight approach was selected in this work to maintain a clear focus on the linear scalarization of the multi-objective problem. Furthermore, it provides essential training stability and implementation simplicity. Empirical results confirm that this fixed parameter is highly effective, providing sufficient support for the algorithm to successfully discover a broad and high-quality Pareto frontier within the specific constraints of our problem setting.

#### 4.1.3. Offloading Action Decision

The UAV-MEC performs the offloading action decision through a scheduler. The scheduler executes the offloading selection action with the maximum Q-value using a greedy probability ϵ, and with probability 1−ϵ, it randomly selects an action from the action space *A*. The action is represented as

(11) $$a_{b,n,t}=\left\{\begin{array}{cc} \underset{a\in A}{\mathrm{argmax}}\;Q\left(s_{b,n,t},a;\theta_{b}^{\prime }\right), & w.p.\;\epsilon ,\\ A\;\mathrm{random}\;\mathrm{action}\;\mathrm{from}\;A, & w.p.\;1-\epsilon , \end{array}\right.$$

where $\max_{a\in A}Q\left(s_{b,n,t},a;\theta_{b}^{\prime }\right)$ represents the target value computed by the target network after receiving the current state $s_{b,n,t}$ through the deep neural network.

#### 4.1.4. Parameter Training and Update

The UAV-MEC trains the parameters $\theta_{b}$ through a training network, which adopts the same deep neural network architecture as the target network. The training network is responsible for real-time parameter learning, while the parameters $\theta_{b}^{\prime }$ of the target network are periodically updated. Specifically, the system accumulates historical experiences of environment states and rewards to form an experience replay buffer $\left[s_{b,n,t},a_{b,n,t},r_{b,n,t},s_{b,n,t+1}\right]$, which provides training data for the training network. The training network outputs the predicted $Q(s_{b,n,t},a;\theta_{b})$ based on the data sampled from the experience replay buffer and optimizes the parameters $\theta_{b}$ using the loss function.

The loss function of the model is defined as follows:

(12) $$Loss\left(\theta \right)={\left[r_{b,n,t}+\gamma \cdot \max_{a_{b,n,t+1}\in A}Q\left(s_{b,n,t+1},a_{b,n,t+1};\theta_{b}^{\prime }\right)-Q\left(s_{b,n,t},a_{b,n,t};\theta_{b}\right)\right]}^{2}$$

where γ is the reward discount factor, and $r_{b,n,t}$ represents the offloading selection reward value.

### 4.2. Algorithm and Optimization Design

In this paper, we design the MORL-LAPB algorithm for computational task offloading. The algorithm aims to balance multiple objectives—minimizing offloading latency, maximizing cluster operation duration, and enhancing power efficiency—while making optimal offloading decisions. The detailed pseudocode is presented in Algorithm 1 [23,24].
**Algorithm 1** MORL-LAPB Algorithm**Require:** 
The arrival task set ${\left\{N_{b,t}\right\}}_{b\in B,t\in T}$, Full Energy of UAV-MEC $\left\{C_{b}^{f}\right\}$, Reward weights $\left\{w_{1},w_{2},w_{3}\right\}$**Ensure:** 
The offloading decision $x_{b,n,t}\in \left\{0,1\right\}$1:Initialize variables: episode←0, actionStep←0, trainStep←02:**for** episode=1,2,…,E **do**3:    **for** each time slot t∈T **do**4:        **for** all MEC b∈B in parallel **do**5:           **if** $C_{t,b}\leq 0$ **then**                        ▹ Check MEC battery status6:               **break**7:           **end if**8:           **for** each new arrival task $n\in N_{b,t}$ **do**9:               Observe the system state $s_{b,n,t}$10:             Select action $a_{b,n,t}$ based on Equation (11)11:             Solve $x_{b,n,t}$ based on $a_{b,n,t}$12:             Allocate task *n* workload to the selected UAV-MEC13:             Calculate the reward $r_{b,n,t}$ based on Equation (10)14:             Observe the next system state $s_{b,n,t+1}$15:             Store transition tuple $\left(s_{b,n,t},a_{b,n,t},r_{b,n,t},s_{b,n,t+1}\right)$ into Replay Buffer16:               **if** $C_{t,b}\leq 0$ **then**             ▹ Check energy after task execution17:                   **break** task loop18:               **end if**19:               Update processing queue $q_{t}$ and energy $C_{t,b}$20:               actionStep←actionStep+121:               **if** actionStep>200 **and** actionStep%20 = 0 **then**22:                   Sample batch from Replay Buffer23:                   Train model and update parameter $\theta_{b}$24:                   trainStep←trainStep+125:                   **if** trainStep%η = 0 **then**26:                       Update target network parameter $\theta_{b}^{\prime }$27:                   **end if**28:               **end if**29:           **end for**30:        **end for**31:        **if** All MECs are depleted **then**32:           **break** time slot loop33:        **end if**34:    **end for**35:**end for**

In multi-objective computational task offloading problems, potential conflicts often exist among optimization objectives: improving one objective may lead to the degradation of others. Therefore, there is no single solution that can simultaneously achieve global optimality across all objectives. How to effectively balance these competing goals becomes the core challenge in the algorithm design process [25,26].

For the computational task offloading optimization problem, Pareto optimality is defined as follows: Let there be two offloading decisions, *A* and *B*. If the following two conditions hold:

(a) For all objective functions, the performance of *A* is no worse than that of *B*, i.e., $\forall i,f_{i}\left(A\right)\leq f_{i}\left(B\right)$ (assuming the objectives are to be minimized);
(b) There exists at least one objective *j* such that $f_{j}\left(A\right)<f_{j}\left(B\right)$.

Then, decision *A* is said to have Pareto dominance over *B*, denoted as A<B. Within the set of all offloading strategies, if a strategy *P* is not dominated by any other strategy, it is referred to as a non-dominated offloading decision. The set of all such non-dominated decisions forms the Pareto set, and its mapping in the objective space constitutes the Pareto front. Accordingly, the goal of this paper is to identify the Pareto optimal solution set of the reward function and to obtain an approximate solution set that can effectively approximate the true Pareto front.

In this paper, an evolutionary optimization algorithm is employed. Starting from an intelligently initialized population, the population and external repository are evaluated and established through the computing task offloading verification strategy. Through multiple rounds of offspring selection, crossover, and mutation operations, the algorithm iterates continuously until the maximum number of generations ($G_{max}$) is strictly reached. This fixed computational budget ensures a fair performance comparison among different algorithms. Finally, the Pareto optimal solution set and its corresponding frontier for the reward weight parameters of the computing task offloading are obtained. The specific process of the evolutionary multi-objective optimization Algorithm 2 is detailed as follows:
**Algorithm 2** Evolutionary Multi-Objective Optimization Algorithm**Require:** 
Population size $P_{N}$, maximum number of generations $G_{max}$, crossover probability $p_{c}$, mutation probability $p_{m}$**Ensure:** 
Approximate Pareto front solution set $\left\{U_{tb},T_{tb},\eta_{tb}\right\}$1:**Initialization:**2:Randomly generate the initial population of weight parameters $\left\{w_{1},w_{2},w_{3}\right\}$3:**while** stopping criterion not met (i.e., generation $<G_{max}$) **do**4:    Perform reproduction through inheritance, crossover, and mutation to generate the offspring population5:    Merge parent and offspring populations6:    Execute the MORL-LAPB algorithm to compute reward factor weights $\left\{U_{tb},T_{tb},\eta_{tb}\right\}$7:    Conduct non-dominated sorting on $\left\{U_{tb},T_{tb},\eta_{tb}\right\}$8:    Apply environmental selection, elitism preservation, and diversity maintenance to form the next generation9:    Increment generation counter10:**end while**

## 5. Simulation Validation

### 5.1. Simulation Environment Setup

In this study, the UAV computational collaboration cluster consists of five UAV-MEC nodes, each capable of receiving task offloading requests from user terminals with a certain probability. Each UAV-MEC node is equipped with an identical offloading model and performs distributed computation offloading to collaboratively execute computational tasks within the cluster.

**Environment Settings:** To emulate real-world task arrival patterns, the simulation considers a probabilistic task arrival model. At each time step, a task arrives with probability *p*, and its computational length is defined as *L*; if no task arrives, the corresponding workload is set to zero. The default environment parameters used in the experiments are listed in Table 3.
**Training Parameters:** Similarly to [27], to simplify the experimental process, the hyper-parameters for both the Reinforcement Learning (RL) model and the Evolutionary Algorithm (EA) are configured as listed in Table 4.

To ensure the reproducibility of the evolutionary algorithm (Algorithm 2) used for tuning the reward weights, the specific technical implementations and hyperparameters are detailed as follows:

**Encoding Scheme:** We employ real-parameter encoding for the weights, with the search space strictly bounded within [0, 1].
**Crossover Operator:** We utilize Simulated Binary Crossover (SBX) to generate offspring, with a crossover probability of 0.9 and a crossover distribution index of 10.
**Mutation Operator:** Polynomial mutation is applied to introduce genetic diversity, with a mutation probability of 0.2 and a mutation distribution index of 20.
**Population Size:** The population size is set to 60 individuals per generation.
**Selection Mechanism:** A tournament selection method is used to select parent individuals for reproduction. Furthermore, an elitism strategy is incorporated to preserve the highest-performing individuals across generations, thereby preventing the loss of good solutions and ensuring stable algorithmic convergence.
**Performance Metrics:** Following [21,27], the proposed method’s offloading performance is evaluated using the average completion time (Objective 2, denoted as $T_{tb}$). In addition, cluster operational duration (Objective 1, denoted as $U_{tb}$) and cluster energy efficiency ratio (Objective 3, denoted as $\eta_{tb}$) are introduced as supplementary performance indicators for comprehensive evaluation.

It is worth noting that the fundamental RL hyperparameters, such as the discount factor (γ) and the exploration rate decay (ε-decay), were empirically determined. Their values were selected based on established best practices in the deep reinforcement learning literature, combined with our preliminary experiments, which demonstrated robust convergence across various test scenarios. While a comprehensive sensitivity analysis of these baseline hyperparameters is undoubtedly valuable, it falls outside the primary scope of this paper, which focuses on the multi-objective offloading architecture. Therefore, we maintain these parameters as fixed constants in our current evaluation, leaving detailed ablation studies on RL hyperparameter sensitivity for future work.

### 5.2. Baseline Methods

To evaluate the performance of the proposed MORL-LAPB algorithm, three competitive baseline methods are selected for comparison, as described below.

To validate the effectiveness of the proposed MORL-LAPB framework, we compare it with the following three benchmark algorithms:

**Random Single-Edge Server Offloading (RSO) [18]:** The RSO method randomly selects an edge server to process each computation offloading task. This approach represents a classical offloading strategy and is widely adopted as a comparison baseline in MEC-related studies.
**Nearest Single-Edge Server Offloading (NSO) [12]:** The NSO method selects the geographically nearest edge server for each computation task offloading. As another classical offloading scheme, it is frequently used for comparison in MEC performance evaluations.
**Deep Reinforcement Learning-Based Single-Edge Server Offloading (DRLSO) [27]:** The DRLSO method utilizes a deep reinforcement learning framework to select the optimal edge server for each computation task. It achieves state-of-the-art performance among DRL-based offloading strategies in MEC environments.

### 5.3. Experimental Results

To clarify how our evaluation supports the core research motivation—simultaneously optimizing latency, operational duration, and energy efficiency—we assess the framework across three dimensions: convergence, diversity, and performance validation. First, convergence and diversity experiments prove learning stability and solution coverage, ensuring a rich set of Pareto-optimal candidate solutions rather than a local optimum. Second, performance validation tests practical adaptability using four core metrics: offloading delay, cluster task duration, energy efficiency, and success rate. Subsequent sensitivity analyses across diverse environmental parameters empirically demonstrate the algorithm’s robust multi-objective superiority and practical viability.

#### 5.3.1. Convergence Analysis

**Figure 4:**
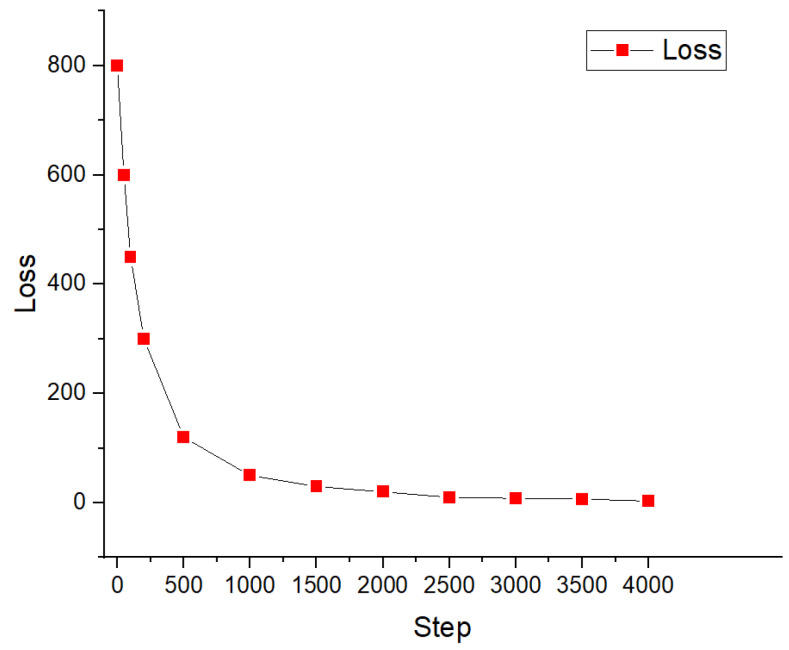
Variation of the training loss during reinforcement learning process.

The loss of the proposed method initially drops rapidly as the training steps increase from 1 to 500, as illustrated in Figure 4. This phenomenon can be attributed to the deep reinforcement learning agent gradually learning to find near-optimal solutions through exploration. Subsequently, the loss continues to decrease quickly and eventually stabilizes at approximately the 1000-step mark. These results clearly demonstrate that the proposed method effectively converges to a stable solution. Furthermore, by recording the execution time of a single iteration, empirical results show that the algorithm consistently completes the computation within 100 ms. This confirms its outstanding computational feasibility and low overhead for both real-time training and deployment.

#### 5.3.2. Diversity

This study employs an evolutionary algorithm to optimize the multi-objective reward parameters $\left(w_{1},w_{2},w_{3}\right)$ through analysis and summarization of the Pareto solution set. The multi-objective reward parameters are defined as real numbers within the range [0, 1], and 10 initial populations are generated. After 10 evolutionary iterations, 9 Pareto-optimal solutions are obtained, and their corresponding objective sets are presented in Table 5.

**Figure 5:**
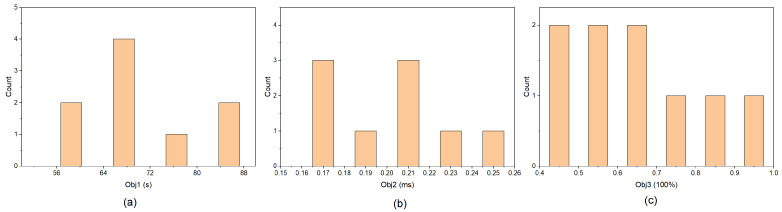
Statistical analysis of the Pareto solution set distribution. (**a**) Distribution of Obj1. (**b**) Distribution of Obj2. (**c**) Distribution of Obj3.

A statistical analysis of the Pareto solution set distribution is presented in Figure 5. As shown in the figure, the objective values are evenly distributed within their respective intervals: Obj1 ranges from [55, 88], Obj2 from [0.16, 0.26], and Obj3 from [0.4, 1.0]. This indicates that the evolutionary algorithm effectively explores the objective space and avoids premature convergence, generating a continuous and stable Pareto front.

**Figure 6:**
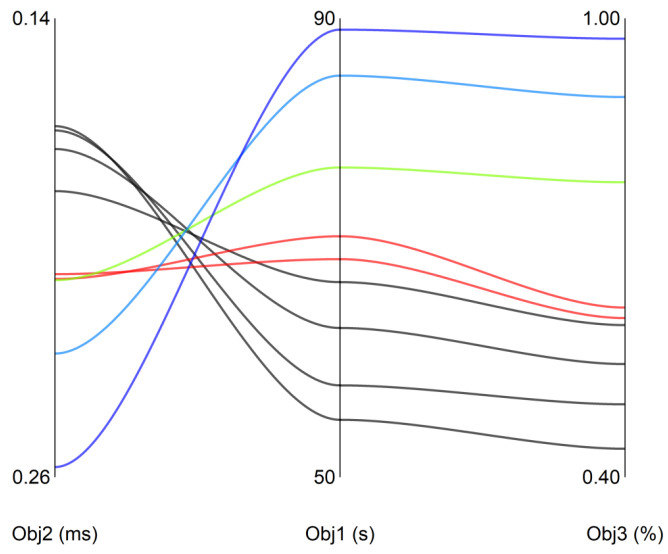
Parallel coordinate plot of multiple objectives. The colored lines are used to highlight representative non-dominated solutions (e.g., extreme solutions favoring a specific objective or a balanced trade-off solution), while the gray lines represent the remaining solutions within the Pareto set.

The distribution is further analyzed using a parallel coordinate plot (Figure 6). Frequent crossings between the lines of Obj1 and Obj2 indicate a strong conflict between them, whereas the near-parallel trends of Obj1 and Obj3 suggest strong synergy.

**Figure 7:**
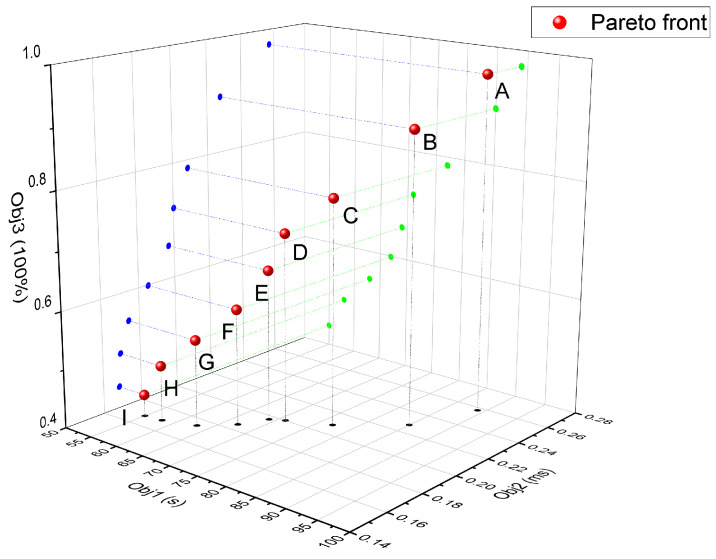
Three-dimensional distribution of the Pareto solution set. The red spheres represent the 3D Pareto front. The letters A through I denote specific representative non-dominated solutions selected for detailed trade-off analysis. The blue, green, and black dots illustrate the 2D projections of these Pareto solutions onto the corresponding coordinate planes (Obj1-Obj3, Obj2-Obj3, and Obj1-Obj2, respectively).

The three-dimensional distribution and its 2D projections (Figure 7) further quantify these trade-offs. For instance, comparing extreme point A and point B implies that sacrificing just 4.5% of Obj1 performance yields an 11% improvement in Obj2. These analyses provide a robust empirical foundation for selecting optimal parameters tailored to specific operational requirements.

#### 5.3.3. Impact of Different Task Processing Deadlines

Comparative experiments were conducted between the proposed method and the baseline methods under various settings.

**Figure 8:**
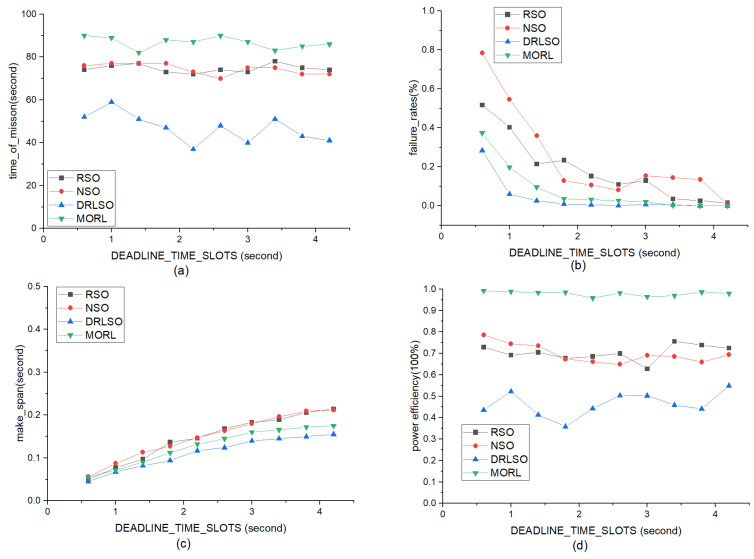
Comparison of various methods (RSO, NSO, DRLSO, and MORL-LAPB) as the task processing deadline varies linearly from 0.6 to 4.2. (**a**) Impact of different deadlines on task duration; (**b**) Impact of different deadlines on offloading failure rate; (**c**) Impact of different deadlines on offloading delay; (**d**) Impact of different deadlines on energy efficiency.

To systematically evaluate the impact of temporal constraints, the task processing deadline was varied from 0.6 to 4.2 (Figure 8). Longer deadlines naturally reduced failure rates across all methods. However, MORL-LAPB maintained the longest and most stable cluster task duration (ranging between 80 and 96) and the highest energy efficiency across all settings. At deadlines >3.8, both MORL-LAPB and DRLSO achieved zero failures. MORL-LAPB’s slightly higher delay compared to baselines reflects its successful processing of difficult tasks that traditional non-learning methods simply dropped.

#### 5.3.4. Impact of Varying Numbers of Arriving Tasks

**Figure 9:**
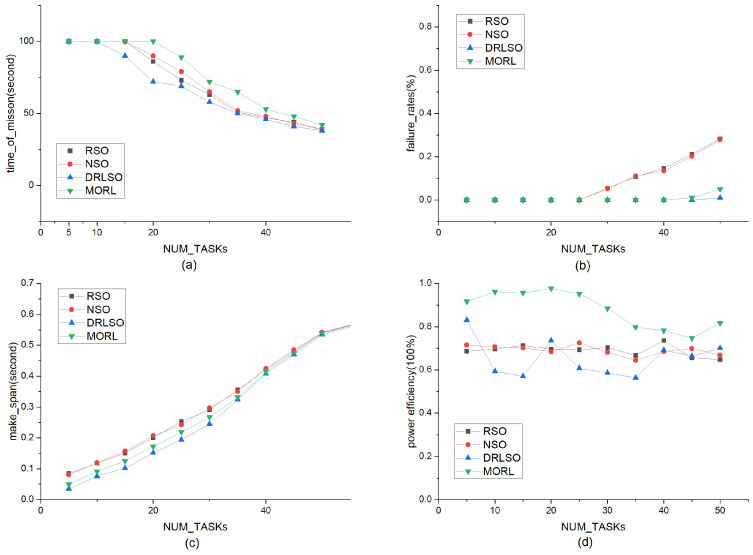
Comparison of various methods (RSO, NSO, DRLSO, and MORL-LAPB) as the task arrival rate varies linearly from 5 to 50. (**a**) Impact of different task arrival rates on task duration; (**b**) Impact of different task arrival rates on offloading failure rate; (**c**) Impact of different task arrival rates on offloading delay; (**d**) Impact of different task arrival rates on energy efficiency.

To evaluate scalability and robustness under heavy traffic loads, the number of arriving tasks $N_{b,t}$ was varied from 5 to 50 (Figure 9). Under increasing workloads, all methods experienced performance degradation, but MORL-LAPB exhibited the slowest decline. At a moderate load (20 tasks), it sustained a full-cycle duration, while baselines fell to 72–90. At a heavy load (35 tasks), MORL-LAPB maintained a duration of 65, significantly outperforming the others (50–52). Failure rates remained near zero for MORL-LAPB and DRLSO, whereas RSO and NSO surged after 25 tasks. Energy efficiency remained consistently highest for MORL-LAPB throughout the spectrum.

#### 5.3.5. Impact of Dynamic Energy Consumption of Computing Units

**Figure 10:**
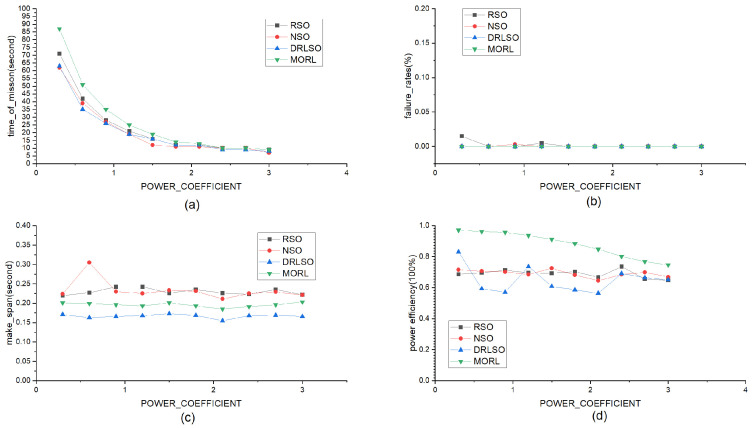
Comparison of various methods (RSO, NSO, DRLSO, and MORL-LAPB) as the dynamic energy consumption rate of computing units varies linearly from 0.3 to 3. (**a**) Impact of different dynamic energy consumption rates on task duration; (**b**) Impact of different dynamic energy consumption rates on offloading failure rate; (**c**) Impact of different dynamic energy consumption rates on offloading delay; (**d**) Impact of different dynamic energy consumption rates on energy efficiency.

To investigate the sensitivity to hardware energy characteristics, the dynamic energy consumption unit cost, $Q_{d}$, was varied from 0.3 to 3.0 (Figure 10). Higher costs nonlinearly reduced operational duration across all schemes. Nevertheless, MORL-LAPB retained the longest durations (e.g., 87 at low cost vs. 62–71 for baselines). It maintained zero failures and the highest energy efficiency throughout, making a slight, justifiable delay trade-off against DRLSO to prioritize global cluster survival and mitigate the “wooden barrel effect.”

#### 5.3.6. Summary of Experimental Results

Overall, the experimental outcomes demonstrate that MORL-LAPB consistently outperforms baseline methods across diverse scenarios. By adaptively balancing energy consumption and service quality, it effectively addresses the conflicting multi-objective demands formulated in our research motivation, providing a highly robust and scalable solution for UAV-MEC cluster computation offloading.

## 6. Discussion

### 6.1. Interpretation of Results and Comparison with Existing Works

The primary objective of this study was to address the multi-objective conflict in UAV-MEC clusters, specifically balancing low-latency service requirements with the limited and heterogeneous energy resources of UAVs. The experimental results presented in Section 5 validate the effectiveness of the proposed MORL-LAPB framework.

Consistent with previous studies using Deep Reinforcement Learning (DRL) for edge offloading, such as DRLSO [27], our method significantly outperforms heuristic approaches (RSO and NSO) in terms of offloading failure rate and average delay. This superiority stems from the ability of the neural network to learn the complex mapping between high-dimensional system states (task queue, channel conditions, energy levels) and optimal offloading decisions, avoiding the load imbalance issues inherent in the “nearest-first” (NSO) or “random” (RSO) strategies.

However, a distinctive finding of this work is the trade-off achieved between cluster operational duration and service latency. While DRLSO achieves low latency, it often neglects the energy variance among UAV nodes, leading to the premature depletion of heavily utilized nodes. In contrast, MORL-LAPB explicitly incorporates the energy balance deviation ($\partial_{b,n,t}$) into the reward shaping mechanism. As observed in Figure 8d and Figure 10d, this strategy allows for MORL-LAPB to maintain the highest cluster energy efficiency and operational duration across various deadlines and energy consumption rates. This implies that sacrificing a negligible amount of latency (as shown in the Pareto front analysis in Figure 7) can yield substantial gains in system sustainability, a critical feature for emergency rescue or long-term monitoring missions where battery replacement is difficult.

Beyond the comparative performance, the robustness of MORL-LAPB is further validated through its sensitivity to key environmental parameters, as detailed in Section 5.3.

**Task Deadlines ($\tau_{n}$):** As shown in Figure 8, even under tight deadline constraints (low $\tau_{n}$), our method maintains a lower failure rate compared to baselines. This indicates that the latency-aware reward component effectively guides the agent to prioritize urgent tasks.
**Task Load (N):** In high-traffic scenarios (Figure 9), while all methods experience degradation, MORL-LAPB exhibits the slowest rate of performance decline. This robustness is attributed to the load-balancing mechanism (e.g., queue length $q_{t,b}$), which prevents specific nodes from becoming bottlenecks during congestion.
**Dynamic Computing Energy Cost ($L_{b,t}$): **Figure 10 demonstrates that the framework adapts well to varying hardware energy specifications. By dynamically sensing the energy consumption rate $L_{b,t}$, the algorithm shifts the optimization focus towards energy conservation when the cost of computing increases, thereby preserving the cluster’s operational lifespan.

Collectively, these results confirm that the proposed algorithm is not sensitive to specific parameter tuning but adapts robustly to diverse operational conditions.

### 6.2. Mechanism Analysis: Why MORL-LAPB Works

The success of MORL-LAPB can be attributed to two core components: the reward-shaping-based RL agent and the evolutionary optimization mechanism. First, the transformed single-objective reward function allows for the agent to perceive the long-term impact of current actions on the cluster’s “barrel effect”—where the system’s lifespan is determined by the UAV with the lowest battery. By penalizing actions that exacerbate energy imbalance, the algorithm naturally guides tasks to nodes with sufficient residual energy, even if they are not geographically closest. Second, the evolutionary algorithm effectively explored the weight parameter space ($w_{1},w_{2},w_{3}$). The linear and non-linear relationships revealed in the Pareto solution set (Figure 7) demonstrate that the conflict between energy efficiency and delay is not static. The proposed framework provides flexibility, allowing for operators to select different weight combinations from the Pareto front to prioritize either speed (for delay-sensitive tasks) or longevity (for endurance tasks) without retraining the model structure.

### 6.3. Limitations

Despite the promising results, this study has certain limitations that should be acknowledged:

**Scalability:** The current approach employs a centralized training architecture. As the number of UAVs increases significantly (e.g., massive swarms), the state space dimension will grow exponentially, potentially leading to the “curse of dimensionality” and making convergence difficult.
**Communication Overhead:** We assumed reliable communication for state information exchange. In practical, highly dynamic environments, exchanging state information (queue length, battery level) between UAVs and the central scheduler incurs signaling overhead and latency, which were simplified in this simulation.
**Mobility Model:** The current study focuses primarily on computation offloading, with UAV hovering positions assumed to be relatively stable during task execution slots. The joint optimization of flight trajectory and task offloading was not fully explored in this specific framework.

### 6.4. Future Research Directions

Based on the findings and limitations discussed above, future research directions include:

**Multi-Agent Reinforcement Learning (MARL):** To address scalability, the centralized framework can be extended to a decentralized MARL approach (e.g., MADDPG or MAPPO), allowing for UAVs to make local decisions while cooperating to achieve global energy balance.
**Joint Trajectory and Offloading Optimization:** Integrating UAV trajectory control into the action space would allow for the system to physically approach user clusters to improve channel quality, thereby reducing transmission energy consumption alongside computational energy optimization.
**Real-World Prototype Validation:** Moving from simulation to hardware-in-the-loop (HIL) testing or small-scale field experiments using varying UAV platforms (e.g., DJI Matrice series with onboard Jetson modules) to validate the algorithm’s robustness under real-world interference and battery characteristics.

### 6.5. Synergy of Evolutionary Optimization and Reinforcement Learning

As a powerful global search paradigm, Evolutionary Optimization (EO) can effectively mitigate the local convergence issues faced by traditional RL methods in multi-objective problems when deeply integrated with Reinforcement Learning (RL). In Multi-Objective Reinforcement Learning (MORL), reward signals are often sparse, and the inherent conflicts between objectives create numerous local optimal traps. While traditional gradient-based methods excel at local search, their greedy pursuit of short-term cumulative rewards makes them susceptible to these traps, severely limiting exploration diversity. EO alleviates this limitation by maintaining a population of candidate policies. Instead of focusing on the iterative improvement of a single policy, it explores disparate regions of the parameter space in parallel. This population-based approach inherently maintains diversity; even if some individuals converge prematurely to a local optimum, others continue to explore uncharted regions. Consequently, EO provides MORL with a global perspective that transcends local gradient information. It not only assists the algorithm in escaping local optima but also enhances the robustness and adaptability of the learned policies in dynamic environments. Recent research, such as the ReinforceAdapt framework, further corroborates this by demonstrating that combining RL with multi-objective evolutionary algorithms enables adaptive operator selection in complex dynamic environments, thereby simultaneously optimizing both convergence and diversity [28].

## 7. Conclusions

In this paper, we proposed MORL-LAPB, a novel multi-objective reinforcement learning framework designed to address the challenge of task offloading strictly constrained by computational and energy resources on UAV cluster. The core idea is to use deep reinforcement learning to achieve a balanced optimization across multiple objectives between offloading latency, task duration, and cluster efficiency and Search for the Pareto optimal solution by using the optimization algorithm component. This study confirms that MORL-LAPB can obtain Pareto fronts over a wide range, providing various trade-off solutions among multiple objectives and satisfying heterogeneous UAV cluster preferences regarding offloading latency, operational time, and cluster efficiency. This study also has certain limitations: the parameters were not partially fixed, and the computational load was huge during the training of the multi-objective evolutionary algorithm. In the future, we plan to adaptively match all parameters of the algorithm model to further verify its practicality and reliability, and explore new ideas to improve our method.

## Figures and Tables

**Table 1:** Summary and comparison of related works on UAV-MEC computation offloading. The checkmark (✓) indicates that the specific objective is considered in the referenced work, while the dash (–) indicates it is not.

Literature	Methodology	Objectives			Advantages	Limitations
		**Lat.**	**Ene.**	**Bal.**		
Wang [15], Liu [16], Pervez [17]	Convex Opt., Game Theory	–	✓	–	Theoretically rigorous with optimality guarantees; suitable for static environments.	High computational complexity; difficult to adapt to highly dynamic environments.
Tang [13], Liu [14], Wang [18], Chen [19]	Q-learning, DQN, DDPG, AC, D3QN	✓	✓	–	Capable of handling high-dimensional continuous state spaces; enables real-time decisions.	Often single-node decision making lacking cluster coordination; struggles to balance conflicting objectives.
Gao [20]	MADDPG	✓	✓	–	Explicitly models multi-UAV interaction and coordination; suitable for distributed clusters.	High training complexity; focuses on individual energy, ignoring cluster energy distribution.
Sun [22]	BCD + SCA	✓	✓	–	Combines traditional optimization with heuristics to achieve effective objective trade-offs.	Offline optimization limits real-time capabilities; requires iterative solving with high overhead.
**Ours (MORL-LAPB)**	**Hybrid Evolutionary-MORL**	✓	✓	✓	**Multi-objective balancing strategy with adaptive Pareto search; prevents “wooden barrel effect”.**	**Slightly higher training complexity, but hyperparameter tuning is automated via EA.**

**Table 2:** Summary of key symbols used in this work.

Symbol	Description
T	The set of time slots.
B	The set of UAV-MECs.
$N_{b,t}$	The set of tasks arriving at UAV-MEC b∈B during time slot t∈T.
Δ	The length of each time slot t∈T.
$\tau_{n}$	The deadline for processing task $n\in N_{b,t}$.
$d_{n}$	The data size of task $n\in N_{b,t}$.
$\rho_{n}$	The computation density of task $n\in N_{b,t}$.
*x*	The decision variable for workload allocation.
*v*	The transmission rate between UAV-MECs.
*f*	The computation capacity of a UAV-MEC.
$T_{b,n,t,b^{\prime }}^{o}$	The processing delay of task $n\in N_{b,t}$ that arrives at UAV-MEC b∈B and is offloaded to UAV-MEC $b^{\prime }\in B$ during time slot t∈T.
$T_{b,n,t}^{s}$	The processing make-span of task $n\in N_{b,t}$ arriving at UAV-MEC b∈B during time slot t∈T.
$L_{b,t,b^{\prime }}$	The power consumption of UAV-MEC *b* at time slot t∈T.
$C_{t},\;C_{f}$	$C_{t}$ denotes the battery energy state of a UAV-MEC at time *t*, while $C_{f}$ represents the battery capacity.
$Q_{d},\;Q_{s}$	$Q_{d}$ refers to the dynamic power consumption of the UAV-MEC computing unit, while $Q_{s}$ denotes the static power consumption.

**Table 3:** Environmental parameters.

Parameter	Value	Parameter	Value
*B*	5	*T*	100
$N_{b,t}$	25	$d_{n}$	[10, 30] Mbits
Δ	0.1 s	$f_{b^{\prime }}$	[10, 50] GHz
$\rho_{n}$	[100, 300] cycles/bit	$\tau_{n}$	1 s
$v_{b^{\prime },b,t}$	[50, 100] Mbit/s	$p_{n}$	0.3
$C_{b}^{f}$	6000 Wh	$C_{0,b}$	[0, 2000] Wh
$Q_{s}$	[5, 10] W	$Q_{d}$	3 W/GCycles

**Table 4:** Parameter settings for algorithms.

Category	Parameter	Value
Reinforcement Learning	Learning rate	0.0005
	Reward decay (γ)	0.2
	Exploration rate (ϵ)	0.99
	ϵ increment	0.0005
	Batch size	64
	Target update interval	200
	Network layers	4
Evolutionary Algorithm	Population size ($P_{N}$)	60
	Max generations ($G_{max}$)	10
	Crossover prob. ($p_{c}$)	0.9
	Mutation prob. ($p_{m}$)	0.2

**Table 5:** Pareto solution set.

No.	Obj1	Obj2	Obj3	Remark	No.	Obj1	Obj2	Obj3	Remark
1	55	0.16823	0.43721	I	6	71	0.20018	0.72175	D
2	58	0.16933	0.49521	H	7	77	0.20849	0.78549	C
3	63	0.17421	0.54796	G	8	85	0.22769	0.89698	B
4	67	0.18521	0.59875	F	9	89	0.25735	0.97312	A
5	69	0.19693	0.65820	E					

## Data Availability

The data presented in this study are available on request from the corresponding authors due to restrictions on privacy and confidentiality, which prevent open sharing of the dataset.
